# A review on cutting-edge innovations in dental implantology

**DOI:** 10.6026/973206300200757

**Published:** 2024-07-31

**Authors:** Naseemoon Shaik, Sai Shilpa Gondyala, Venkatesh Bejagam, Yerra Sindhuri, Shifa Fathima Shajahan, Tejashree Suhas Bhavsar

**Affiliations:** 1 Department of Pedodontics and Preventive Dentistry, MNR Dental College and Hospital, Sangareddy, Telangana, India; 2 Sri Sai College of Dental Surgery, Telangana, India; 3 Department of Prosthodontics Crown and Bridge, Meghna Institute of Dental Sciences, Nizamabad, Telangana, India; 4 Periodontics, GSL Dental College and Hospital, Andhra Pradesh, India; 5 Department of Periodontics, Sathyabama Dental College and Hospital, Chennai, India; 6 Department of Prosthodontics Crown and Bridge, HKES's S.N Dental College, Karnataka, India

**Keywords:** Dental implants, osseo-integration, implant materials, surgical techniques, digital technology, pediatric dentistry, 4D printing

## Abstract

Dental implantology has advanced significantly with innovations in materials, designs, surgical techniques, and digital technology aimed at enhancing patient outcomes. Applications span specialties like prosthodontics, periodontics, orthodontics, oral surgery, aesthetic dentistry, and pediatric dentistry, with pediatric implants showing a success rate of 90-95% over ten years. 4D printing is emerging as a transformative technology for dynamic implants. This review traces the historical development from ancient attempts to modern titanium-based designs, emphasizing milestones like osseo-integration discovery by Dr. Per-Ingvar Brånemark. Recent progress includes enhanced titanium surfaces, biocompatible zirconia, and composite materials, as well as innovations in implant design such as nanotechnology-enhanced surfaces and personalized implants, all aimed at improving osseointegration and stability, while surgical approaches now prioritize minimally invasive techniques, immediate loading protocols, and computer-guided procedures to minimize patient discomfort and expedite recovery was discussed.

## Background:

Dental implants have revolutionized the field of restorative dentistry by providing a superior solution for replacing missing teeth compared to traditional prosthetic options like bridges or dentures. Their popularity stems from significant benefits such as enhanced aesthetics, improved functionality that mimics natural teeth, and increased patient comfort and satisfaction [1]. Despite their success, traditional implantology approaches are not without limitations, including potential complications such as peri-implantitis and challenges in achieving optimal bone integration in certain clinical scenarios [2]. In response to these challenges, ongoing innovations in dental implant technology have been pivotal. These advancements aim to address existing limitations while enhancing the overall efficacy and longevity of dental implants. Therefore, it is of interest to explore and analyze the latest developments in dental implant materials, design modifications, surgical techniques, and digital technologies.

## Brief history of dental implants:

## Early attempts:

The concept of dental implants dates back to ancient civilizations. Archaeological evidence shows that ancient Egyptians and Mayans used materials such as shells and stones as rudimentary dental implants. These early attempts were not functionally successful but laid the foundation for future developments [1].

## 20^th^ century breakthroughs:

In the 1950s, Dr. Per-Ingvar Brånemark, a Swedish orthopedic surgeon, made a ground breaking discovery that fundamentally changed the field of implantology. During his research on blood flow in rabbit bone, he used titanium chambers to study microcirculation. Upon completion of his experiments, he found it difficult to remove the titanium chambers from the rabbit's bone. Further investigation revealed that bone tissue had grown so closely around the titanium that it effectively adhered to the metal without any intervening soft tissue layer. This phenomenon surprised Dr. Brånemark, as it contradicted the prevailing belief at the time that metal could not integrate with living bone. Dr. Brånemark termed this natural phenomenon "osseointegration," derived from the Latin words "osseo" meaning bone and "integration" meaning to make whole. He hypothesized that the titanium metal had formed a direct structural and functional connection with the living bone tissue, a process that was previously unknown and revolutionary in its implications for medical and dental implants [2]. The process of osseointegration involves several key steps: Initially, upon implantation of a titanium implant into the bone, a series of molecular events occur at the implant surface. Blood proteins rapidly adsorb to the implant's surface, initiating a cascade of cellular responses. Osteoblasts, specialized bone-forming cells, migrate to the implant surface and begin to deposit new bone matrix directly onto the titanium [3]. Over time, this newly formed bone tissue matures and mineralizes, firmly anchoring the implant within the jawbone. Dr. Brånemark's discovery of osseointegration laid the foundation for modern dental implantology, establishing titanium as the material of choice due to its biocompatibility and ability to promote bone integration. Today, osseointegration remains a cornerstone in implant dentistry, ensuring the long-term success and stability of dental implants for millions of patients worldwide [4]. Key factors influencing osseointegration include implant material biocompatibility, surface characteristics (such as roughness or bioactive coatings), and surgical techniques that minimize trauma to bone and soft tissues. Titanium and its alloys are commonly used due to their excellent biocompatibility and ability to promote osseointegration. Surface modifications enhance the implant's integration capability, while surgical precision and loading protocols also impact the success of osseointegration [3].

## Evolution of techniques and materials:

Throughout the latter half of the 20th century, significant advancements were made in both the materials used for implants and the techniques for their placement. The introduction of various surface treatments and designs improved the stability and longevity of implants. Innovations such as acid etching, sandblasting, and plasma spraying enhanced the osseointegration process, making implants more reliable [5].

## Digital era:

The advent of digital technology in the late 20^th^ and early 21^st^ centuries has further transformed dental implantology. Digital imaging, computer-aided design/manufacturing (CAD/CAM), and 3D & 4D printing have enabled more precise planning and customization of implants, improving outcomes and patient satisfaction.

## Innovations in implant materials:

## Titanium and titanium alloys:

Titanium remains the gold standard for dental implants due to its biocompatibility and strength. Recent innovations focus on improving the surface of titanium implants to enhance osseointegration. Methods such as acid etching, sandblasting, and plasma spraying have been developed to modify the implant surface, promoting better integration with bone tissue [6].

## Zirconia implants:

Zirconia is becoming a popular alternative to titanium because of its aesthetic properties and biocompatibility. Zirconia implants offer a natural tooth color, making them ideal for patients with thin gingival biotypes or concerns about metal allergies. Advances in zirconia processing have improved its mechanical properties, making it a viable option for dental implants [7].

## Composite materials:

Composite materials, which combine ceramics with polymers, are being explored for their potential to offer the benefits of both materials. These composites aim to provide the strength of ceramics and the flexibility of polymers, potentially reducing stress on surrounding bone and improving long-term outcomes [6, 8].

## Innovations in implant design:

## Surface modifications:

Surface modifications remain a critical area of innovation. Nanotechnology has enabled the creation of Nano patterned surfaces that mimic natural bone, enhancing cell attachment and proliferation. These nanostructures improve Osseo integration rates and may reduce healing times [9].

## Tapered and threaded designs:

Implant designs have evolved to include tapered and threaded structures that mimic the natural shape of tooth roots. These designs enhance primary stability, especially in soft bone conditions, and distribute occlusal forces more evenly, reducing the risk of implant failure [10].

## Custom-made implants:

Advances in 3D printing and computer-aided design/manufacturing (CAD/CAM) technologies have enabled the production of custom-made implants tailored to individual patient anatomy. Customization ensures a better fit, potentially reducing surgical time and improving patient outcomes.

## The role of 4D printing in implants:

## Concept and mechanism:

4D printing is an emerging technology that adds the dimension of time to 3D printing. In 4D printing, materials are designed to change shape or properties over time in response to external stimuli such as temperature, moisture, or pH [11]. This dynamic capability offers potential applications in dental implantology.

## Innovations in surgical techniques:

## Minimally invasive surgery:

Minimally invasive surgical techniques, such as flapless surgery and guided implant placement, have been developed to reduce patient discomfort and accelerate recovery times. These techniques use detailed preoperative planning and advanced imaging technologies to precisely place implants with minimal tissue disruption [12].

## Immediate loading:

Immediate loading protocols, where prosthetic components are attached to implants shortly after placement, are gaining popularity. Advances in implant stability and surface technology have made immediate loading a viable option, reducing treatment times and improving patient satisfaction [13].

## Computer-guided surgery:

Computer-guided surgery uses detailed imaging and planning software to create precise surgical guides. These guides enhance the accuracy of implant placement, reduce surgical errors, and improve outcomes. This technology is particularly beneficial in complex cases and for patients with compromised bone structure [14].

## Applications of implants in various specialty fields in dentistry:

## Prosthodontics:

In prosthodontics, dental implants are used to replace single or multiple missing teeth and to support dentures. Implant-supported prostheses offer enhanced stability, function, and aesthetics compared to traditional removable dentures. Innovations such as all-on-four techniques, where four implants support full-arch prosthesis, have revolutionized treatment options for edentulous patients [15].

## Periodontics:

Periodontists often use dental implants to replace teeth lost due to periodontal disease. Implants can help preserve the jawbone and maintain the structural integrity of the oral cavity. Additionally, periodontal therapy often involves preparing the site for implant placement by addressing bone and soft tissue deficiencies [16].

## Orthodontics:

Dental implants can serve as temporary anchorage devices (TADs) in orthodontics. TADs provide stable anchor points for moving teeth into desired positions without relying on patient compliance or adjacent teeth for support. This application allows for more precise and efficient orthodontic treatments [17].

## Oral and maxillofacial surgery:

Oral and maxillofacial surgeons frequently place dental implants, especially in complex cases involving significant bone loss or anatomical challenges. Advanced imaging and surgical planning tools have improved the precision and success rates of implant surgeries. Bone grafting and sinus lift procedures are commonly performed to create a suitable foundation for implants [18, 19].

## Aesthetic dentistry:

In aesthetic dentistry, dental implants play a crucial role in restoring the natural appearance of the smile. Innovations in implant design and materials have led to more lifelike restorations that blend seamlessly with the patient's natural teeth. Immediate implant placement and provisionalization techniques allow for quicker aesthetic improvements [20].

## Pediatric dentistry:

Although dental implants are less commonly used in pediatric dentistry, they can be a viable option for children and adolescents who have lost teeth due to trauma, congenital absence, or other reasons. Implants in pediatric patients require careful consideration of growth and development. Research indicates that implants placed in young patients can have high success rates when proper protocols are followed [17, 21]. Assessing skeletal growth, bone quality, oral health, and selecting appropriate implants and surgical techniques, along with immediate loading protocols, post-operative care, and regular follow-up, are essential for successful dental implant placement in young patients [22, 23, 24-25]. By meticulously following these protocols, clinicians can enhance the predictability and success rates of dental implant treatments in young patients, supporting their long-term oral health and quality of life [26, 27-28]. Each step is tailored to mitigate risks, promote healing, and maximize the functional and aesthetic outcomes of dental implant therapy. According to a recent study, the success rate of dental implants in pediatric patients is approximately 90-95% over a 10-year period, provided that the implants are monitored closely and adjusted as necessary to accommodate jaw growth and development [21]. These implants help in maintaining arch space, preserving bone, and improving aesthetics and function in growing patients.

## Applications in dental implants:

## Customized healing abutments:

4D printing can be used to create healing abutments that change shape to adapt to the healing gingival tissue, promoting optimal soft tissue contours and aesthetics. This adaptability can lead to better long-term outcomes in terms of tissue health and implant stability [11].

## Smart implants:

4D-printed smart implants can respond to changes in the oral environment, such as mechanical stress or microbial presence. These implants could potentially release therapeutic agents in response to infection or inflammation, enhancing the healing process and reducing the risk of complications [28].

## Shape-memory alloys:

Shape-memory alloys (SMAs) are a class of materials used in 4D printing that can return to their original shape after deformation when exposed to specific stimuli. SMAs can be used to create implants that adjust their shape post-placement, ensuring a better fit and integration with the bone [26].

## Conclusion:

The field of dental implantology is continuously evolving, with cutting-edge innovations enhancing the effectiveness, safety, and aesthetics of dental implants. Advances in materials, implant design, surgical techniques, and digital technologies are transforming clinical practice, offering improved outcomes for patients. The incorporation of 4D printing in dental implants represents a frontier with the potential to further revolutionize the field. As research and development continues the future of dental implantology promises even greater strides in delivering optimal patient care.
